# Motor scores, therapeutic hypothermia and neurological outcome after cardiac arrest

**DOI:** 10.1186/cc12255

**Published:** 2013-03-19

**Authors:** M Davidson

**Affiliations:** 1 Plymouth Hospitals NHS Trust, Plymouth, UK

## Introduction

Accurate prediction of neurological outcome after cardiac arrest is desirable to prevent inappropriate withdrawal of life-sustaining therapy in patients who could have a good neurological outcome, and to limit active treatment in patients whose ultimate neurological outcomes are poor. Established guidelines to predict neurological outcome after cardiac arrest were developed before the widespread use of therapeutic hypothermia. The American Association of Neurology guidelines [1] currently recommend that absent or extensor motor scores on day 3 post arrest are reliable indicators or poor neurological outcome with a false positive rate of 0 to 3%.

## Methods

A review of existing literature was undertaken to examine whether the utility of motor scores to predict poor neurological outcome is influenced by the use of therapeutic hypothermia.

## Results

Six studies were identified [2-7] that investigated the use of motor scores on day 3 post cardiac arrest in patients who had received therapeutic hypothermia. False positive rates (defined as 1 - specificity) for predicting poor neurological outcome were calculable in five of the six studies [2-6] and were 14%, 24%, 11%, 25% and 12% respectively. In all studies the FPR for motor scores of extension or worse were significantly higher than the 0% (0 to 3% 95% CIs) in the AAN guidelines.

## Conclusion

Motor scores at day 3 post cardiac arrest of extension or worse do not reliably predict poor neurological outcome when therapeutic hypothermia has been used. Clinical neurological findings may not be valid predictors of poor neurological outcome after therapeutic hypothermia.
